# Three Dimensional-Arterial Spin Labeling Evaluation of Improved Cerebral Perfusion After Limb Remote Ischemic Preconditioning in a Rat Model of Focal Ischemic Stroke

**DOI:** 10.3389/fnana.2022.893953

**Published:** 2022-06-30

**Authors:** Tianxiu Zheng, Xiaolan Lai, Jiaojiao Lu, Qiuyan Chen, Dingtai Wei

**Affiliations:** ^1^Department of Radiology, Ningde Municipal Hospital Affiliated to Ningde Normal University, Ningde, China; ^2^Department of Hematology, Ningde Municipal Hospital Affiliated to Ningde Normal University, Ningde, China; ^3^Department of Central Laboratory, Ningde Municipal Hospital Affiliated to Ningde Normal University, Ningde, China

**Keywords:** LRP, 3D-ASL, VEGF, CBF, AIS, CD31

## Abstract

**Purpose:**

To investigate the application value of 3D arterial spin labeling (3D-ASL) for evaluating distal limb ischemic preconditioning to improve acute ischemic stroke (AIS) perfusion.

**Materials and Methods:**

A total of 40 patients with AISs treated in our hospital from January 2020 to December 2020 were recruited, and 15 healthy individuals who were examined in our hospital during the same period were included as the control group; all of these participants were scored on the National Institutes of Health Stroke Scale (NIHSS) and examined by MRI. Sequences included conventional sequences, diffusion-weighted imaging (DWI), magnetic resonance angiography (MRA), and 3D-ASL, and cerebral infarct volume and cerebral blood flow (CBF) in the area of the infarct lesion were measured. After 3 months of treatment, patients with AIS were scored on the modified Rankin Scale (mRS) and divided into good prognosis and poor prognosis groups. In total, 55 adult male Sprague–Dawley rats were divided randomly into three groups: 20 in the middle cerebral artery occlusion (MCAO) group, 20 in the MCAO + limb remote ischemic preconditioning (LRP) group, and 15 in the sham group. In total, 48 h after the procedures, conventional MRI, DWI, and 3D-ASL sequence data were collected, and 2,3,5-trphenyltetrazolium chloride monohydrate (TTC) staining and behavioral scoring were performed. CBF was recorded in the infarct lesion area and the corresponding contralateral area, and the affected/contralateral relative values (rCBF) were calculated to compare the differences in rCBF between different groups. The pathological changes in brain tissues were observed by HE staining, and the expression of vascular endothelial growth factor (VEGF) and platelet endothelial cell adhesion molecule-1 (PECAM-1/CD31) in brain tissues was detected by immunofluorescence and real-time quantitative polymerase chain reaction (RT-qPCR). The protein expression of VEGF was detected by western blotting.

**Results:**

Hypertension and internal carotid atherosclerosis are high-risk factors for ischemic stroke, and CBF values in the infarct area are significantly lower than those in the corresponding areas on the contralateral side. NIHSS and mRS scores and CBF values have higher specificity and sensitivity for the prognosis of patients with AIS. LRP significantly reduces the infarct area, improves behavioral deficits in rats with cerebral ischemia, reduces neurological injury and histological damage, protects vascular structures, and promotes neovascularization. In addition, 3D-ASL showed a significant increase in brain tissue perfusion in the ischemic area after LRP, and the expression of VEGF and CD31 showed a significant positive correlation with CBF values.

**Conclusion:**

Three dimensional (3D) ASL can be used to evaluate LRP to improve stroke perfusion, and its protective effect may be closely related to LRP-induced vascular regeneration.

## Introduction

Stroke is a common clinical condition, and the incidence of ischemic stroke is as high as 80%, mainly caused by occlusion or stenosis of the blood supply to brain tissue (Moretti et al., 2015).

A large number of studies have confirmed that limb remote ischemic preconditioning (LRP) has a clear ischemic protective effect (Du et al., 2020), but its mechanism is still unclear (Ren et al., 2015; Liang et al., 2019; Basalay et al., 2020). Vascular endothelial growth factor (VEGF), as a potent growth factor with neuroprotective and angiogenic effects, has become a potential therapeutic target (Wang et al., 2020).

Imaging is a common auxiliary examination method for cerebrovascular diseases and can assist in the diagnosis of diseases through structural brain imaging and functional brain imaging. Cerebral blood flow (CBF) provides an immediate response to the hemodynamic changes in brain tissue, and cerebral perfusion imaging is the main method for obtaining CBF currently. Conventional perfusion imaging requires injection of contrast or radiotracer and is invasive to the patient. With the continuous development of technology, new imaging techniques have been introduced (Shang et al., 2018). The three-dimensional (3D) arterial spin labeling (3D-ASL) is a new rapid, non-invasive, contrast-free perfusion MRI technique that reflects the perfusion level of brain tissue by CBF (Wong et al., 2014; Zhang et al., 2015). The most important advantage of this method is the capability of reproducibly quantitative measurement of CBF values *in vivo* without the introduction of external contrast agents, making this method outperform than traditional contrast push technique and strikingly useful in reflecting the physiopathologic alterations of the cerebral blood (Cutajar et al., 2014; Naresh et al., 2016). Currently, it is mainly used to assess cerebrovascular lesions (Liu et al., 2021), neurodegenerative diseases (Joseph, 2021), and tumors as an aid to diagnosis (Kong et al., 2017) and pre-operative assessment (Lu et al., 2017), and no reports have been published on the use of 3D-ASL for evaluating distal limb ischemic preconditioning (IPC) to improve ischemic stroke perfusion.

Therefore, the present study investigated the value of 3D-ASL magnetic resonance cerebral perfusion imaging for evaluating IPC in distal limb ischemia to improve ischemic stroke perfusion and investigated whether the neuroprotective effect of LRP could be achieved by modulating VEGF.

## Materials and Methods

### Animals

**FIGURE 1:**
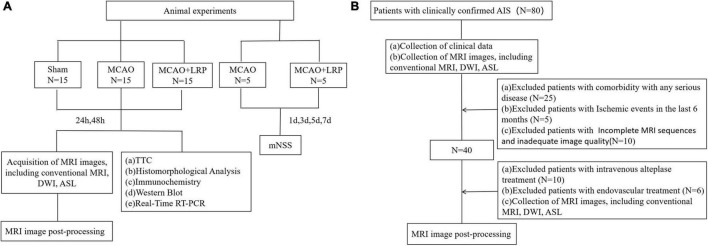
Flowchart. **(A)** Animal experiment flowchart and **(B)** The workflow for patient selection.

Fifty-five healthy adult male Sprague–Dawley rats (260–330 g, 2.5–3 months old) were purchased from Shanghai Slaughter Laboratory Animal Co, Shanghai, China. The rats were kept in a clean environment in a dedicated animal house at a room temperature of 20–25°C and 50–60% humidity. The protocol for the study was approved by the Institutional Animal Care and Use Committee (2018-A19). Animal experiment flowchart is shown in Figure 1A.

### Establishment of a Rat Model of Middle Cerebral Artery Occlusion and the Limb Remote Ischemic Preconditioning Method

The establishment of rat model of MCAO was established based on previous reports (Landman et al., 2019; Peng et al., 2019; Wu et al., 2020). Food was withheld from the rats for 24 h before the modeling procedure, but water was still provided normally. The rats were anesthetized with isoflurane for gas anesthesia: the initial anesthetic induction flow was 5%, and the maintenance flow was 2–3%. The body temperature of the rats was maintained at 37 ± 2°C by applying thermostatic pads throughout the surgery. The rats in the ischemic group were modeled by (1) exposing the femoral artery of the right lower extremity for 90 min before suturing the wound to exclude the effect of the lower extremity incision on the experiment; (2) cutting the skin of the rat’s neck along the median line, isolating the right common carotid artery, external carotid artery, and internal carotid artery, respectively, and inserting a presoaked 2% heparin wire plug (0.26 mm in diameter) from the proximal end of the external carotid artery near the bifurcation The LRP pre-treatment was performed as described by Landman et al. (2019) The femoral artery of the right lower extremity was first isolated and clamped closed with an arterial clip for 15 min and then perfused for 15 min after the arterial clip was released to recover, and the operation was repeated for three cycles, while cerebral ischemia modeling was performed afterward (same method as before). Laser Doppler flowmetry was applied to monitor cerebral perfusion in rats to ensure the validity of the modeling, and a decrease in CBF to more than 75% was considered valid for modeling. It needs to be ensured that the final number of rats modeled meets the grouping requirements aforementioned. The sham-operated group underwent the same surgical operation except for the vascular clamping.

### Patients

This study was approved by the local ethics committee and institutional review board. The requirement of informed consent was waived due to the retrospective nature. The workflow for patient selection is shown in Figure 1B. A total of 40 patients aged 50–80 years with acute ischemic stroke (AIS), some of whom received LRP intervention in advance (Ho et al., 2020), underwent MRI sequences including conventional MRI, diffusion-weighted imaging (DWI), magnetic resonance angiography (MRA), and ASL data from January 2020 to December 2020 were reviewed retrospectively. For all the patients, according to different conditions, the corresponding treatment plan should be selected, such as regular treatment, intravenous alteplase treatment (IVT), and endovascular treatment (EVT) (Yang et al., 2022). Patients who received only regular treatment or received both regular treatment and LRP intervention were assigned to regular treatment and regular treatment + LRP groups, respectively. Exclusion criteria included premorbid dependency [modified Rankin Scale (mRS) score < 3], significant comorbidity with any serious diseases, and a life expectancy under 6 months (Table 1). Other cerebral lesions were also counted as an exclusion criterion. In addition, 15 healthy people who underwent physical examination in our hospital during the same period were included in the control group.

**TABLE 1:** The inclusion and exclusion criteria.

Inclusion criteria	Exclusion criteria
Age: 50–80 years	No clinical intervention treatment
Ischemic stroke confirmed by CT scan	Hemorrhagic stroke on CT scan
<24 h from onset to MRI examination	Fluctuating neurological deficit
	Transient ischemic attack
	Other cerebral lesions: cerebral tumors, arteriovenous malformation
	Ischemic events in the last 6 months (AIS, myocardial infarction, etc.)
	Premorbid mRS > 3
	Comorbidity with any serious disease and life expectancy of <6 months

### Imaging Protocol

All the procedures were performed on a 3.0 T MRI scanner (GE, Discovery MR 750) using an eight-channel head coil. Scanning sequences included (1) 3D-ASL: TR/TE = 4,632 ms/10.5 ms, 3D spiral fast spin echo, spiral sequence acquired with eight spiral arms, 512 points acquired per arm, post-labeling delay time = 1,525 ms, NEX = 3, slice thickness = 2 mm; (2) DWI: TR/TE = 4,000 ms/87.6 ms, matrix = 160 × 160, field of view (FOV) = 240 mm × 240 mm, slice thickness = 5 mm, slice gap = 1.5 mm and two *b*-values (*b* = 0 and 1,000 s/mm^2^); (3) T2-fluid-attenuated inversion recovery (FLAIR): TR/TE = 8,400 ms/145 ms, matrix = 256 × 256, FOV = 240 mm × 240 mm, slice thickness = 5 mm, slice gap = 1.5 mm.

Magnetic resonance scans were performed 48 h after rat modeling, and T2-FLAIR, DWI, and 3D-ASL images were acquired using a 3.0 T MRI scanner (GE, Discovery MR 750) with a mouse four-channel dedicated coil (WK602, Magtron Inc., Silicon Valley, CA, United States). After successful isoflurane anesthesia, the rats were fixed in a prone position on a scanning stand with the central point of the brain and the scan baseline perpendicular to the line connecting the tip of the forebrain to the central point. Continuous inhalation anesthesia with 1.5–2.5% isoflurane was maintained throughout. Scanning sequences included the following: (1) 3D-ASL: TR/TE = 4,444 ms/12.9 ms, 3D spiral fast spin echo, spiral sequence acquired with eight spiral arms, 512 points acquired per arm, post-labeling delay time = 1,000 ms, NEX = 3, slice thickness = 2 mm; (2) DWI: TR/TE = 3,300 ms/2.5 ms, matrix = 128 × 128, FOV = 60 mm × 60 mm, slice thickness = 2 mm, two *b*-values (*b* = 0 and 1,000 s/mm^2^); (3) T2-FLAIR: TR/TE = 8,450 ms/145 ms, matrix = 256 × 256, FOV = 100 mm × 100 mm, slice thickness = 2 mm.

### Magnetic Resonance Data Analysis

The raw data were transferred to a GE ADW4.5 workstation and post-processed by Functool software to obtain the CBF pseudocolor maps of 3D-ASL. The three levels showing the largest ischemic range were selected, and the ischemic area shown in the CBF image was manually outlined as the area of interest. The corresponding CBF was measured, and the contralateral values were obtained by the specular method. The three levels were repeatedly measured three times, and the average values were taken. The relative value (rCBF) was calculated by comparing the CBF values on the ischemic side with those on the contralateral side. On the cranial DWI sequence, each layer of the acute cerebral infarct was outlined separately by the post-processing software, and the area was automatically calculated layer by layer. Finally, the infarct volume was obtained by multiplying the thickness of the layers by the area of the infarct in each layer and summing these values over all layers. In the case of multiple foci, the acute cerebral infarct volumes in the ipsilateral internal carotid artery supply area were added to each other. FLAIR/DWI “mismatch” was used to evaluate the ischemic penumbra (Escalard et al., 2018; Sun et al., 2018). The processing was performed by two neuroradiologists (6 and 8 years of experience), and disagreements were resolved by another neuroradiologist (15 years of experience).

### TTC Staining of Brain Slices

TTC staining assay was performed based on previous report (Yang et al., 1998). After 24 h, the rats were deeply anesthetized with sodium pentobarbital and then sacrificed. The brains were removed and briefly cooled to a temperature of approximately −80°C. The brain was sliced coronally in a 2 mm interval with a brain matrix, for a total of seven coronal sections. The sliced sections were then subsequently stained with 1% TTC (Sigma–Aldrich) at 37°C for 30 min. Finally, the stained sections were transferred into 4% paraformaldehyde at 4°C for fixation. Image Pro-Plus (Version 6.0., Media Cybernetics, Inc., Rockville, MD, United States) was used to calculate the infarct area.

### Neurological Deficit Test

Rats in the LRP and middle cerebral artery occlusion (MCAO) groups were scored behaviorally 48 h after modeling using a four-point scale (Niego et al., 2017): no signs of any neurological deficit were scored as 0; mild neurological deficit (e.g., flexion, elevation, shoulder inversion, and elbow extension of the forelimb contralateral to the lesion when the rat was lifted and suspended by the tail) was scored as 1; moderate focal neurological deficit (signs of rotation to the side of paralysis) was scored as 2 score of 3 was given for severe focal neurological deficit (signs of falling to the opposite side of the lesion); a score of 4 was given for the absence of spontaneous activity and decreased level of consciousness.

### Behavioral Scoring

Another independent group of MCAO and LRP mice was scored on the modified Neurological Severity Scale (mNSS) (Yousefi-Manesh et al., 2021). The mNSS was applied to determine neurological function by a researcher blinded to the experimental groups. First, mNSS was evaluated 24 h after MCAO to identify qualified mice. In total, ten mNSS tests were performed 3, 5, and 7 days after transient MCAO by the same researcher who was blinded to the experimental groups. In addition, mice that died during the tests were recorded carefully.

### Histomorphological Analysis

After 48 h of modeling, the rats were anesthetized by intraperitoneal injection using 10% chloral hydrate, and 200 ml each of 0.9% saline and 4% paraformaldehyde were given in turn by transcardial perfusion. Brain tissues were peeled, and coronal sections were made at two points from the optic chiasm to the occipital lobe, routinely fixed, dehydrated, transparent, impregnated with wax, embedded, and sectioned 3 μm thick. One sheet from each group was taken for routine HE staining. Paraffin sections were dewaxed, dehydrated in an ethanol gradient, stained with hematoxylin and eosin, cleared with xylene, sealed with neutral gum, and observed under light microscopy.

### Immunochemistry

Forty-eight hours after ischemia induction, rats were deeply anesthetized with sodium pentobarbital and transcardially perfused with heparinized saline (0.9%), and the brain was removed and fixed with 4% paraformaldehyde for paraffin embedding. The paraffin blocks were then cut into 10 μm sections (Leica SM2010R sliding microtome, Leica Microsystems Inc., Buffalo Grove, IL, United States) mounted on polylysine-coated glass slides (Thermo Scientific, Waltham, MA, United States), and incubated at 45°C overnight. The sections were deparaffinized by soaking in two changes of xylene for 5 min each and then serially transferred to 100, 90, and 70% ethanol solution for 1 min each. The sections were then rinsed with distilled water for 5 min. For immunofluorescence, antigen retrieval was performed by heating the slides for 30 min in citrate-EDTA buffer containing 10 mM citric acid (pH 6.2), 2 mM EDTA and 0.05% Tween-20. The brain sections were washed with 1 × PBS (3 × 10 min) and blocked in a blocking solution (0.3% Triton X-100 in 1 × PBS +10% serum, which was generated from the species of the secondary antibodies) at room temperature for 1 h. The sections were then incubated in the primary antibody solution at 4°C overnight. After primary incubation, the brain sections were washed in PBS, followed by incubation of Alexa Fluor secondary antibodies for 1 h at room temperature. After a final wash step, the brain sections were mounted in an antifade glass medium with DAPI (Life Technologies, Carlsbad, CA, United States) for image capture. Horseradish peroxidase (HRP) was used for marking, and DAB was used for color rendering in immunohistochemistry.

### Western Blot

Total protein was extracted from cells with RIPA lysis buffer (Beyotime, Shanghai, China) containing protease inhibitors. The protein concentration of the lysates was measured using BCA protein assays (Beyotime, China). In total, forty micrograms of protein were separated on a 10% SDS-polyacrylamide gel and blotted onto polyvinylidene difluoride (PVDF) membranes (Millipore, Billerica, MA, United States). After blocking with 5% bovine serum albumin (BSA) for 1 h, the membranes were incubated with primary antibodies [VEGF (MA532038, Thermo Fisher Scientific, Waltham, MA, United States)] overnight at 4°C and HRP-conjugated secondary antibodies for 1 h at room temperature. Immunoreactive signals were detected using the enhanced chemiluminescence (ECL) detection system. Immunoblotting of tubulin was performed as an internal control.

### RNA Isolation and Real-Time Quantitative Polymerase Chain Reaction

In total, RNA was extracted using TRIzol reagent (Thermo Fisher Scientific, United States). In total, five micrograms of template RNA were used to synthesize the first-strand complementary DNA (cDNA) using a PrimeScript RT reagent kit (Promega, Shanghai, China). Quantitative real-time RT-PCR analysis was performed using SYBR Fast qPCR Mix (Promega, Shanghai, China) and an Applied Biosystems StepOnePlus Real-Time System (Applied Biosystems, Foster city, CA, United States) with SYBR Green to determine the mRNA expression level of a gene of interest. A reference gene, beta-actin (β-actin) was used to normalize gene expression levels. The mRNA abundance was analyzed using the comparative threshold cycle (2^–ΔΔ*CT*^) method. The primers used in the RT-PCR were as follows: VEGF: forward: 5′-GGGCCTCGGTTCCAGAAG-3′; reverse: 5′-GCAGCCTGGGACCACTTG-3′; CD31: forward: 5′-AACAAACTAGCAAGGAGCAGGAAGG-3′; reverse: 5′-CGCT TCGGAGACTGGTCACAATG-3′; β-actin: forward: 5′-CCCG CGAGTACAACCTTCTTG-3′; reverse: 5′-GTCATCCATGGC GAACTGGTG-3′. All the samples were measured in at least three independent experiments, and the results are expressed as the mean ± SD of the comparative analysis.

### Statistical Analysis

All the statistical analyses were performed using SPSS 22.0 for Windows (SPSS, Chicago, IL, United States) and Prism 5.0 software (GraphPad, San Diego, CA, United States). Continuous variables with a normal distribution are expressed as the mean ± SD, and one-way ANOVA was used. Non-normal distributed continuous data were presented as the median (25–75%), the Mann–Whitney *U*-test was employed. The classification variables were represented by percentages and were tested with the χ^2^ test. The correlations of CBF, National Institutes of Health Stroke Scale (NIHSS), and CBF with AD were analyzed by the Pearson correlation analysis. *P* < 0.05 was considered statistically significant.

## Results

### Reduction of Cerebral Infarct Volume, Neurological Deficit, and Cell Damage by Limb Remote Ischemic Preconditioning

**FIGURE 2:**
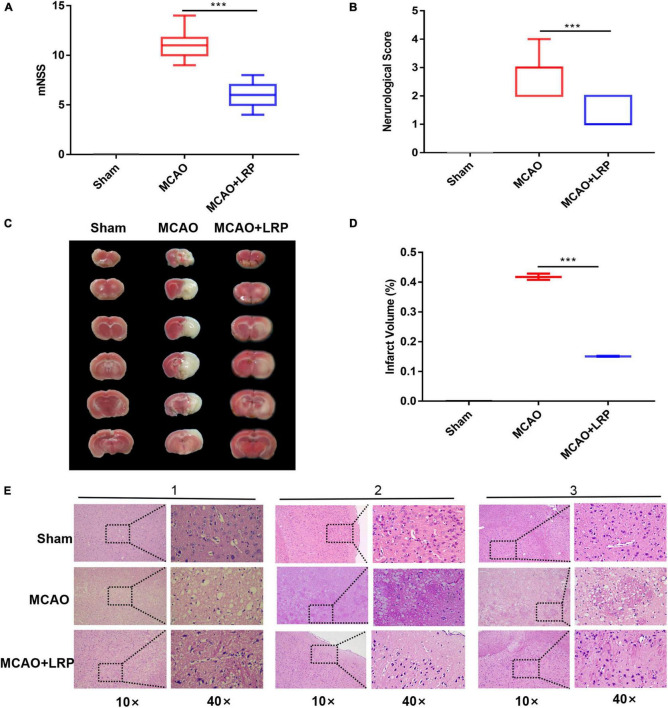
Reduction of cerebral infarct volume, neurological deficit, and cell damage by LRP. **(A)** Modified neurological severity scores of ischemic rats (the data are presented as the mean ± SD, ***P < 0.001). **(B)** Neurological scores of ischemic rats (the data are presented as the mean ± SD, ***P < 0.001). **(C,D)** Representative images of TTC staining and statistical analysis of the brain infarct volume (the data are presented as the mean ± SD, ***P < 0.001). **(E)** Histological morphology in the vicinity of the infarcted area was protected by LRP. Tissue necrosis and mesh-like structure were reduced in the LRP group (magnification, 10× and 40×; scale bar, 50 μm).

After 48 h of ischemic stroke, a significant increase in neurological deficit test scores and mNSS was observed in the MCAO group (2.7 ± 0.64, 11 ± 1.26), which was significantly reduced by MCAO + LRP (1.3 ± 0.46, 5.8 ± 1.21, *P* < 0.001 vs. MCAO; Figures 2A,B). In addition, the infarct volume in the MCAO group was 41.79 ± 0.86%, whereas MCAO + LRP mice displayed significantly reduced infarct volume compared with untreated animals (15.10 ± 0.12%, *P* < 0.001 vs. MCAO; Figures 2C,D). In the sham group, the cells in the infarct area were neatly arranged, the nucleus of each cell was located in the center, and the cell membrane was intact; in the MCAO group, by contrast, the cell arrangement was disordered, the cells were swollen and ruptured, and the nuclei were shrunken. When MCAO and LRP were both performed, the cell structure was normal, the arrangement was intact, and the cell membranes were intact fundamentally (Figure 2E).

### Limb Remote Ischemic Preconditioning Increased the Expression of Vascular Endothelial Growth Factor in the Ischemic Brain 48 h Post-stroke

**FIGURE 3:**
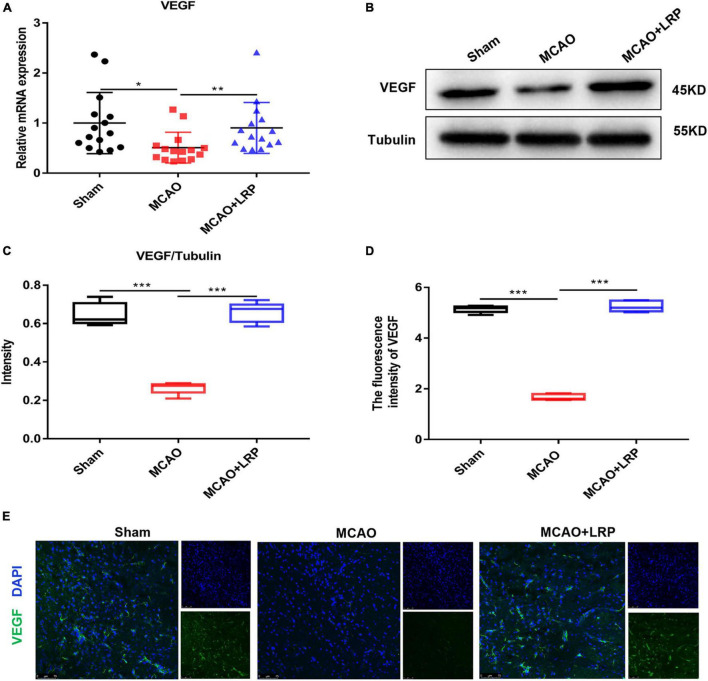
Limb remote ischemic preconditioning (LRP) increased the expression of VEGF in the ischemic brain 48 h post-stroke. **(A)** VEGF mRNA changes were confirmed using RT-PCR. **(B,C)** VEGF expression changes were confirmed using western blot analysis. **(D,E)** The expression of VEGF in the vicinity of the infarcted area was increased by LRP. After treatment with LRP for 48 h, the fluorescence intensity of VEGF was significantly increased (the data are presented as the mean ± SD, * *P* < 0.05; ** *P* < 0.01; ***P < 0.001). Bar = 75 μm.

To test whether LRP could regulate the expression of VEGF in ischemic tissue in rats, we measured VEGF mRNA and protein levels in penumbra tissue. The results of real-time RT-PCR showed that the mRNA expression of VEGF was halved at 48 h post-stroke in the ischemic brain, whereas LRP upregulated VEGF mRNA expression by nearly 50% (Figure 3A). Similarly, the western blotting (WB) results (Figure 3B) and statistical analysis showed that LRP increased the protein expression of VEGF in the ischemic brain 48 h post-stroke (Figure 3C); additionally, the VEGF expression levels were significantly increased in the MCAO + LRP group (Figures 3D,E, *P* < 0.001) compared with the MCAO group.

### Limb Remote Ischemic Preconditioning Promoted Angiogenesis and Enhanced Vessel Density in the Vicinity of the Infarcted Area

**FIGURE 4:**
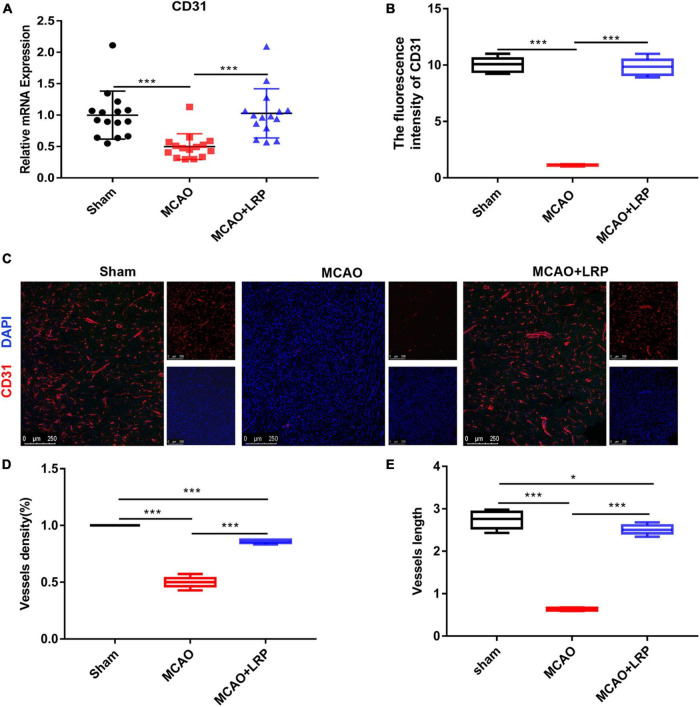
Limb remote ischemic preconditioning (LRP) promoted angiogenesis and enhanced vessel density in the vicinity of the infarcted area. **(A)** CD31 mRNA changes were confirmed using RT-PCR. **(B–E)** The vessel density and vessel length in the vicinity of the infarcted area were increased by LRP. After treatment with catalpol for 48 h, the LRP group showed a significant increase in vessel number and length per area (the data are presented as the mean ± SD, *P < 0.05, ***P < 0.001). Bar = 250 μm.

Vascular damage poses a critical threat to tissue survival after recanalization in focal cerebral ischemia by disrupting the integrity of the blood–brain barrier and leading to microcirculatory clogging. Vascular remodeling is a promising therapeutic strategy for ischemic stroke. To observe angiogenesis near the infarcted area, we used CD31 to label vessels. The real-time RT-PCR results showed that the mRNA expression of CD31 was decreased 48 h post-stroke in the ischemic brain, whereas LRP upregulated CD31 mRNA expression by nearly 50% (Figure 4A, *P* < 0.001). Compared with those in the sham group, vessel density and vessel length in the MCAO group were decreased significantly. However, after treatment with LRP for 48 h, vessel density and vessel length were significantly increased (Figures 4B–E, *P* < 0.001).

### Clinical and Radiographic Characteristics of Patients

**FIGURE 5:**
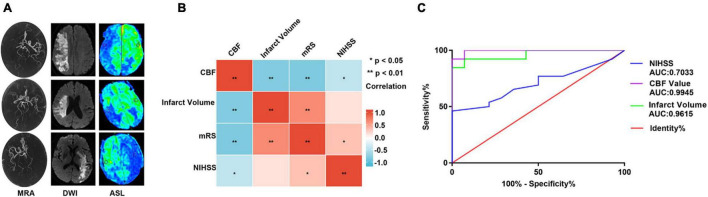
Clinical and radiographic characteristics of patients. **(A)** Magnetic resonance imaging in patients with AIS, including MRA, DWI, and CBF maps. **(B)** Correlation analysis of CBF value, cerebral infarct volume, mRS, and NIHSS (the depth of the color block represents the size of the correlation coefficient, and * represents statistical significance). **(C)** Receiver operating characteristic (ROC) curves of NIHSS, cerebral infarction volume, and CBF on the prognosis of patients with AIS. ** *P* < 0.01.

The clinical and radiographic characteristics of the 40 patients are shown in Table 2. AIS significantly correlated with arterial hypertension, carotid atherosclerosis, or CBF (all *P* < 0.001). There were significant differences in terms of NIHSS scores, CBF, and infarct volume between patients with good prognosis and those with poor prognosis (*P* = 0.037, *P* < 0.0001, and *P* < 0.0001, respectively), as shown in Table 3. Figure 5A shows magnetic resonance images of three typical AIS patients, including MRA, DWI, and CBF maps. In a correlation analysis of CBF, cerebral infarct volume, mRS, and NIHSS, all pairs of variables were correlated except that NIHSS was not correlated with cerebral infarct volume (Figure 5B). Binary logistic regression analysis showed that CBF yielded the greatest area under the curve (0.995) in differentiating between good prognosis and poor prognosis after AIS (Figure 5C).

**TABLE 2:** Clinical and radiographic characteristics of patients.

Characteristics (total *n* = 55)	NC (*n* = 15)	AIS (*n* = 40)	*P*-value
Age, years	66.4 ± 6.46	68.15 ± 11.06	0.231
Male sex, *N* (%)	5 (33.33%)	20 (50%)	0.277
BMI, kg/m^2^	22.36 ± 3.21	22.63 ± 2.71	0.781
**Medical history**			
Arterial hypertension, *N* (%)	6 (40.0%)	37 (92.5%)	*P* < 0.001
Diabetes mellitus, *N* (%)	1 (6.67%)	10 (25%)	0.256
Atrial fibrillation, *N* (%)	1 (6.67%)	4 (10.0%)	0.708
Alcohol consumption, *N* (%)	2 (13.3%)	2 (5.0%)	0.633
Smoking, *N* (%)	2 (13.33%)	3 (7.50%)	0.231
Carotid atherosclerosis, *N* (%)	6 (40.0%)	39 (97.5%)	0.001
Depression, *N* (%)	1 (2.50%)	1 (6.67%)	0.471
**Clinical and laboratory findings at admission**			
Systolic BP, mmHg	135.93 ± 25.42	163.53 ± 17.71	0.001
Diastolic BP, mmHg	79.60 ± 11.50	91.48 ± 12.96	0.003
Heart rate, bpm	75.40 ± 10.26	74.0 ± 12.11	0.693
Temperature, °C	36.39 ± 0.23	36.38 ± 0.28	0.959
Total cholesterol, mmol/L	4.63 ± 0.90	5.02 ± 1.63	0.388
Triglycerides, mmol/L	1.49 ± 0.89	1.56 ± 1.00	0.814
**Imaging findings**			
Infarct volume, cm^3^	–	22.55 ± 10.23	
Cerebral blood flow, ml/100 g/min	35.99 ± 2.23	18.80 ± 4.65	*P* < 0.001

**TABLE 3:** Analysis of indicators related to patient prognosis.

	NIHSS	CBF	Infarct volume
Good prognosis (*n* = 14)	4.00 (2.00–7.50)	21.59 (20.35–26.12)	11.82 (11.02–13.22)
Poor prognosis (*n* = 26)	8.50 (3.50–11.25)	16.68 (13.90–18.34)	24.70 (21.82–37.32)
*P*	0.037	*P* < 0.001	*P* < 0.001

*Data were presented as the median (25–75%).*

*NIHSS, National Institutes of Health Stroke Scale; CBF, cerebral blood flow.*

### Limb Remote Ischemic Preconditioning Improved Blood Perfusion in the Region of Cerebral Infarction

**FIGURE 6:**
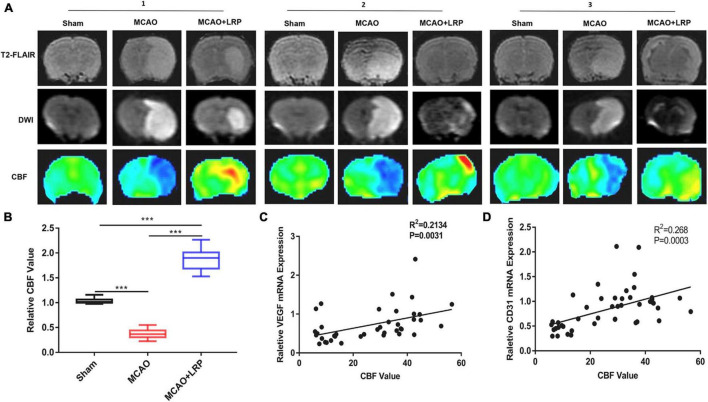
Limb remote ischemic preconditioning (LRP) improved blood perfusion in the region of cerebral infarction. **(A,B)** T2-FLAIR, CBF maps, and DWI images were matched, and perfusion in the vicinity of the infarcted area was increased by LRP. After treatment with LRP for 48 h, the LRP group showed a significant increase in the CBF value (the data are presented as the mean ± SD, ***P < 0.001). **(C,D)** Correlation analysis between the mRNA expression levels of VEGF, CD31, and CBF values.

Increasing CBF and alleviating cerebral ischemia effectively protected neurons in MCAO rats. To observe CBF near the infarcted area, we used 3D-ASL. Forty-eight hours after ischemic stroke, a significant reduction in CBF was observed in the MCAO group (6.51 ± 0.21), which was significantly increased by MCAO + LRP (51.80 ± 4.23, *P* < 0.001 vs. MCAO; Figures 6A,B). The correlation analysis between the mRNA expression levels of VEGF and CD31 and the CBF values are shown in Figures 6C,D. Relative VEGF mRNA expression, relative CD31 mRNA expression, and CBF values were positively correlated (*R*^2^ = 0.2134, *P* < 0.0031, and *R*^2^ = 0.268, *P* < 0.0003, respectively). Meanwhile, 3D-ASL was used to determine the changes in CBF near the infarction area in patients with AIS, and it was found that LRP could improve cerebral perfusion in patients with AIS (Table 4, *P* < 0.001).

**TABLE 4:** Clinical and radiographic characteristics of patients with different treatment options.

Characteristics (total *n* = 24)	Regular treatment (*n* = 11)	Regular treatment + LRP (*n* = 13)	*P*-value
Age, years	67.64 ± 12.01	66.31 ± 13.57	0.804
Male sex, *N* (%)	5 (45.45%)	7 (53.85%)	0.688
BMI, kg/m^2^	20.85 ± 2.58	23.50 ± 3.52	0.051
Arterial hypertension, *N* (%)	10 (90.91%)	12 (92.31%)	0.358
Diabetes mellitus, *N* (%)	4 (36.36%)	1 (7.70%)	0.092
Atrial fibrillation, *N* (%)	1 (9.09%)	2 (15.38%)	0.649
Alcohol consumption, *N* (%)	1 (9.09%)	–	0.277
Smoking, *N* (%)	2 (18.18%)	–	0.116
Carotid atherosclerosis, *N* (%)	11 (100%)	13 (100%)	1
Prior treatment CBF	18.53 ± 3.07	19.56 ± 4.23	0.511
Post-treatment CBF	15.71 ± 4.19	28.20 ± 2.54	*P* < 0.001

*LRP, limb remote ischemic preconditioning; CBF, cerebral blood flow.*

## Discussion

In the present study, we found that CBF had higher sensitivity and specificity than NIHSS scores or infarct volume in the prognosis of patients with AIS. LRP reduced cerebral infarct size and improved neurological function in rats after ischemic stroke, while CBF measurements reflected a corresponding improvement in perfusion. Our results suggest that 3D-ASL can be used to assess the improvement of post-stroke perfusion by LRP, and its protective effect may be closely related to LRP-induced vascular regeneration.

Acute ischemic stroke is a pathological process involving multiple mechanisms. Vascular wall lesions and hemodynamic changes are the main factors in the pathogenesis of ischemic stroke (Thon and Jovin, 2021; Zhang et al., 2021), which is consistent with our results. On the basis of atherosclerosis, hemodynamic changes, cerebral perfusion, decreased embolus clearance and decreased establishment of collateral circulation in the ischemic area, brain cells become hypoxic and apoptotic, forming a central necrotic area and a surrounding ischemic penumbra (Yousefi-Manesh et al., 2021); this process is accompanied by excessive release of free radicals, intracellular calcium overload, and excitatory amino acid toxicity, resulting in neuronal damage (Catanese et al., 2017; Uzdensky, 2019). Therefore, neuroprotective therapy is particularly important in the treatment of AIS.

Ischemic preconditioning refers to the ability of mild ischemic pre-stimulation to increase the resistance of a target organ to subsequent ischemia, preventing severe injury; this induced protective effect is called remote ischemic preconditioning (RIPC) if it occurs between different anatomically distant organs. Although early RIPC studies were related to the heart, the protective effects of RIPC have been demonstrated in multiple organ systems and shown to be effective. Various organs, including the kidneys (Long et al., 2022), lungs (Zheng et al., 2020), liver (Wu et al., 2021), stomach (Filaretova et al., 2021), and brain (Pignataro, 2021), also respond to brief ischemia exposures with an increased resistance to severe ischemia. LRP is one of the most commonly used pre-processing methods today (Wei et al., 2012). In a prior study, LRP protected against ventricular arrhythmia caused by total ischemia and reperfusion, and it ultimately reduced the incidence of sudden cardiac death by suppressing JNK activation and restoring Cx43 expression (Yan et al., 2021). LRP in the context of AIS in rats is safe, reduces infarct size, and improves functional recovery (Basalay et al., 2020). In addition, LRP may have a protective effect against ischemic stroke-induced brain injury through microRNA-144 downregulation of PTEN and upregulation of Akt (Zhong et al., 2021). The results of the present study also showed that LRP mitigated the degree of neurological deficits, reduced infarct size, and improved the cytological morphology of the ischemic injury area in rats with AIS.

As an important component of the neurovascular units in the brain, the vasculature is a key initiator and important therapeutic target in many diseases, such as stroke and Alzheimer’s disease. The main pathological features of ischemic stroke are vascular injury and the resulting neuronal damage, such as decreased capillary density and loss of neuronal apoptosis, leading to tissue hypoxia and neurological dysfunction in the infarcted hemisphere (Skvortsova et al., 2011). In the early stages of ischemic stroke, functional damage to the penumbra tissue is somewhat reversible. Maintaining the structural and functional integrity of blood vessels near the infarcted area, promoting vascular neovascularization, and restoring blood supply to the ischemic penumbra are keys to avoiding the aggravation of brain tissue damage and are important therapeutic strategies for ischemic stroke (Jahan and Vinuela, 2009).

Revascularization is considered as an important process to improve the blood supply around the infarct area and promote neurological recovery after ischemic stroke. In the present study, we found that after 48 h of cerebral ischemia, the vascular structure of the infarct area was disturbed: the vessels were missing their normal tubular structure. However, the LRP group showed reduced perivascular tissue damage and relatively intact vascular tubular structures. In addition, LRP increased the vascular density near the infarct area in the ischemic rats. Angiogenesis is mainly induced by hypoxia and inflammation and is regulated in a precise and orderly manner through the interaction of multiple factors and signaling pathways (Wang et al., 2020), and VEGF is a key regulator of the angiogenic process (Abdulkadir et al., 2022). This study shows that LRP can upregulate the expression of VEGF protein and mRNA and upregulate the expression of CD31 mRNA, which regulates and promotes functional angiogenesis, resulting in neural remodeling.

The 3D-ASL technique uses hydrogen protons in arterial blood as an endogenous tracer and spin-labels hydrogen protons in the source region of blood flow in the scanning plane. The labeled hydrogen protons cause a change in the longitudinal relaxation T1 of the local tissue in the scan region, resulting in a flip-labeled image, and the labeled hydrogen proton flow is proportional to the perfusion of the brain, resulting in a perfusion-weighted image, i.e., a CBF map (Calamante et al., 1999). Several prior studies indicated that the NIHSS score by itself, and in conjunction with diagnostic imaging, has been shown to have a positive predictive value in determining the functional outcome (Campbell et al., 2015; Goyal et al., 2015; Haranhalli et al., 2019). In contrast, our study found that mRS was closely associated with infarct volume, NIHSS, and CBF and that CBF had the highest specificity and sensitivity for the prognosis of AIS. The 3D-ASL has a high-negative predictive value (NPV) in assessing AIS and patient prognosis when patients present with acute stroke symptoms, which DWI and angiography cannot provide (Buch et al., 2021). Several studies have investigated the use of 3D-ASL to detect perfusion abnormalities in the context of infarction (Chen et al., 2016; Noguchi et al., 2016). Lu et al. (2021) suggested that hyperperfusion on 3D-ASL correlated with successful recanalization and might be an independent prognostic marker for good neurological outcomes in patients with AIS. 3D-ASL can objectively show the development of collateral circulation around the infarcted area and provide an informed assessment of the disease and prognosis (Yuan et al., 2015). In this study, we evaluated the effect of LRP with the aid of 3D-ASL and showed that the infarcted area of the rat brain was significantly hypoperfused, whereas LRP significantly increased the perfusion of brain tissue in the ischemic area. The results suggest that the 3D-ASL technique has unique advantages for evaluating LRP to improve ischemic stroke perfusion.

The sample size of this study was small because of the short onset of acute stroke, which was difficult to monitor within the effective time. In the later stage, we hope to further explore the clinical application value of 3D-ASL by expanding the sample size and investigating the protective mechanism of LRP in depth.

## Data Availability Statement

The original contributions presented in this study are included in the article/supplementary material, further inquiries can be directed to the corresponding author/s.

## Ethics Statement

The studies involving human participants were reviewed and approved by Ningde Municipal Hospital Affiliated to Ningde Normal University. Written informed consent for participation was not required for this study in accordance with the national legislation and the institutional requirements. The animal study was reviewed and approved by Ningde Municipal Hospital Affiliated to Ningde Normal University. Written informed consent was obtained from the individual(s) for the publication of any potentially identifiable images or data included in this article.

## Author Contributions

TZ wrote the manuscript. TZ, QC, and JL completed the data collection. TZ and XL completed the data statistics and the subject was completed under the guidance of DW. All authors read and approved the final manuscript.

## Conflict of Interest

The authors declare that the research was conducted in the absence of any commercial or financial relationships that could be construed as a potential conflict of interest.

## Publisher’s Note

All claims expressed in this article are solely those of the authors and do not necessarily represent those of their affiliated organizations, or those of the publisher, the editors and the reviewers. Any product that may be evaluated in this article, or claim that may be made by its manufacturer, is not guaranteed or endorsed by the publisher.
